# Managing Acute Behavioural Disturbances in the Emergency Department Using the Environment, Policies and Practices: A Systematic Review

**DOI:** 10.5811/westjem.2017.4.33411

**Published:** 2017-05-15

**Authors:** Tracey J. Weiland, Sean Ivory, Jennie Hutton

**Affiliations:** * St Vincent’s Hospital Melbourne, Emergency Practice Innovation Centre, Melbourne, Victoria, Australia; † St Vincent’s Hospital Melbourne, Department of Emergency Medicine, Melbourne, Victoria, Australia; ‡ The University of Melbourne, Melbourne School of Population and Global Health, Neuroepidemiology Unit, Victoria, Australia; § The University of Melbourne, Faculty of Medicine, Dentistry and Health Sciences, Melbourne, Victoria, Australia

## Abstract

**Introduction:**

Effective strategies for managing acute behavioural disturbances (ABDs) within emergency departments (EDs) are needed given their rising occurrence and negative impact on safety, psychological wellbeing, and staff turnover. Non-pharmacological interventions for ABD management generally fall into four categories: environmental modifications; policies; practice changes; and education. Our objective was to systematically review the efficacy of strategies for ABD management within EDs that involved changes to environment, architecture, policy and practice.

**Methods:**

We performed systematic searches of CINAHL Plus with Full Text, PsycINFO, MEDLINE, and EMBASE, as well as reference lists of relevant review articles to identify relevant studies published between January 1985 – April 2016. We included studies written in English, which reported management of behavioural disturbances in adults associated with the ED through the use of environmental modifiers (including seclusion, restraint, specialised rooms, architectural changes), policy, and practice-based interventions excepting education-only interventions. Efficacy outcomes of interest included incidence, severity, and duration of ABD, incidence of injuries, staff absenteeism, restraint use, restraint duration, and staff and patient perceptions. Two reviewers independently screened titles and abstracts, and assessed the relevancy and eligibility of studies based on full-text articles. Two authors independently appraised included studies. A narrative synthesis of findings was undertaken.

**Results:**

Studies reporting interventions for managing ABDs within the ED are limited in number and quality. The level of evidence for efficacy is low, requiring caution in conclusions. While there is preliminary evidence for environmental change in the form of specialised behavioural rooms, security upgrades and ED modifications, these are not supported by evidence from controlled studies. Many of these “common sense” environmental changes recommended in many guidelines have been widely implemented in EDs.

**Conclusion:**

There is an unambiguous gap in the literature regarding the efficacy of interventions for ABD management in EDs involving environmental, policy or practice-based changes. With growing demand on EDs, and with increasing numbers of ABDs, identification of robust evidence-based interventions for safe and effective ABD management is vital.

## INTRODUCTION

Violence, aggression and abuse are highly prevalent in the healthcare sector, and have had a rising incidence over the past 15 years.^1^–^7^ This is despite the widespread requirement that workers have the right to a safe and harassment-free workplace.^8^ Together, violence and aggression can be conceptualised within the broader definition, “acute behavioural disturbance” (ABD). ABDs include verbal abuse, threats, physical assaults, assaults with bodily fluids and aggressive behaviours.^1^,^7^ An ABD describes a person’s conduct that does not respond to normal verbal intervention and interrupts the daily workings of the hospital department.^7^,^9^ ABDs affect the morale, physical and psychological wellbeing of staff and staff performance, and, therefore, the healthcare provided to patients.^10^

A major focal point for ABDs within the healthcare sector is within emergency departments (EDs). EDs have the highest reports of violence globally.^2^,^11^–^13^ EDs are generally open 24/7 and serve a large population of various backgrounds. In the United Kingdom, a staff survey identified that >30% of ED staff were assaulted.^13^ Although unacceptably high, these figures may be significantly underestimated due to widespread underreporting.^2^,^14^ Nonetheless, it is clear that minimising the frequency and impact that ABDs have within EDs is critical.

A significant body of research has identified factors leading to ABDs within the ED and other hospital units. ABDs can be conceptualised as arising due to patient factors, staff factors, environmental factors and their interaction.^15^ It is logical, therefore, that efforts to reduce and effectively manage ABDs would be aimed at each of these areas. A Cochrane review^16^ is currently underway examining the effectiveness of education and training interventions to prevent and minimise aggression toward healthcare workers. An examination of non-pharmacological methods other than educational interventions for staff is lacking. An integrative review by Anderson et al., focusing on interventions to reduce violence against emergency nurses reported in publications between 1986–2007, revealed a lack of substantial robust evidence for ABD management interventions.^17^,^18^ Despite this, the use of environmental modifiers, such as specialised rooms^19^–^24^ and changes in policy and practice, is becoming common. While the present paper reviews the efficacy of non-pharmacological management methods for ED ABDs, particularly focusing on policy, practice and environmental interventions, we take a broader focus to the study by Anderson et al. by expanding inclusion criteria to all ED staff, instead of predominately ED nurses. When used in conjunction with other topical literature, the findings may assist in guiding practice, interventions and management of ABDs within the ED.

### Aims and Objectives

Our goal was to systematically search, summarise and critically appraise primary literature regarding efficacy of non-pharmacological strategies to manage ABDs within EDs, focused on environmental, architectural, policy or practice-based interventions. Efficacy studies were considered those that assessed changes in incidence, duration or severity of ABDs, incidence of injuries, staff absenteeism, restraint use, restraint duration, or subjective staff or client perceptions.

**Population Health Research Capsule**

What do we already know about this issue?

Acute behavioural disturbances are common occurrences in emergency departments and represent a threat to safety and wellbeing. Non-pharmacological management strategies fall into four categories: educational interventions for staff; changes to policy or practice; or environmental modification. A systematic review of educational interventions is underway but a thorough examination of other non-pharmacological methods is lacking.

What was the research question?

Is the management of acute behavioural disturbances in emergency departments using non-pharmacological methods including changes to policy, practice or environment efficacious?

What was the major finding of the study?

The quality of all studies reviewed was weak. There is little evidence suggesting that the acute behavioural disturbance management strategies reviewed are efficacious. An unambiguous gap exists in the literature and there is a strong need to balance tailored interventions with unified approaches suitable for implementation on a widespread scale.

How does this improve population health?

This study underscores the need for rigorous testing of efficacy of interventions to manage acute behavioural disturbances. Continued practice of non-pharmacological strategies should be undertaken alongside rigorous evaluation.

## METHODS

### Criteria for considering studies for this review

Studies were eligible if they did the following: (a) included adult participants (aged > 18 years) associated with the ED including service users/patients, staff, visitors and police; (b) were concerned with managing ABDs within the ED; (c) involved environmental, physical or architectural management strategies, policy interventions, and new practices; (d) assessed any outcome measures of incidence, duration, or severity of ABDs, incidence of injuries, staff absenteeism, frequency or duration of restraint use, and staff or client perceptions; (e) were randomised control trials, non-randomised controlled trials, prospective or retrospective cohort studies, case-control studies, or pre-post observational studies; (f) were written in English; (g) were full-text articles; and (h) were published between January 1, 1985, and April 21, 2016. This date range was selected to overlap with previous systematic reviews including that by Nelstrop,^18^ which was restricted to seclusion and restraint, but included studies set both in the ED and other acute inpatient settings; and Anderson,^10^ who undertook an integrative review of methods for managing ED violence but restricted it to studies of nurses.

We excluded studies if they used qualitative methods only, were integrated literature reviews, systematic reviews or meta-analyses. In light of a systematic review currently underway on educational interventions for clinicians to better manage ABD,^16^ we excluded studies reporting educational interventions only. Control groups (including pre-intervention), had to involve standard care.

### Search of literature strategy

We conducted electronic database searches of OVID MEDLINE, CINAHL Plus with Full Text, PsycINFO (via OVID) and EMBASE (via OVID) on April 21, 2016, using Boolean/phrase, free-text search strategies, and medical subject heading (MeSH). Searches within titles and descriptors were used (Appendix A–D).

We also searched reference lists of meta-analyses, systematic reviews, and integrated reviews for relevant articles.

### Study selection

Titles and abstracts were reviewed by two independent authors (TW, SI) to determine relevance. Full texts of potentially relevant articles were then evaluated against inclusion criteria. Disagreements between reviewers were resolved by consensus; if no agreement could be reached, the opinion of a third author (JH) was planned to be determinant.

### Quality appraisal

Quality of included studies was appraised by two independent reviewers (TW, JH), unblinded to study purpose, using the Effective Public Health Practice Project (EPHPP) Quality Assessment Tool For Quantitative Studies (Hamilton Tool).^25^ Reviewers resolved disagreements by discussion and consensus.

### Data extraction and synthesis

Data extraction was completed independently by one author (SI) and verified by another (TW) and included the following: primary author (year); setting, country; study design; participants; length of study; participant characteristics; interventions or exposures; outcome measures; main findings; study limitations.

Heterogeneity of interventions, data and methodologies meant statistical pooling was unsuitable; a narrative synthesis was undertaken.

## Results

### Search results

The systematic search resulted in 4,708 articles (Figure). We removed 1,940 duplicates, leaving 2,768 for review. Review of titles and abstracts of articles excluded 2,736. Full texts were sought for 35 articles written in the English language. Of these, three articles could not be sourced despite extensive searches by a librarian and multiple attempts to contact authors. Available full texts were further assessed against the inclusion criteria to provide a total of eight relevant articles^26^–^31^ (Table 1).

### Description of studies

Included studies were mostly interrupted time series (n=5). One study used an analytic cohort design, one was a prospective cohort study (single group pre-post), and there was one (non-randomised) control trial (Table 1).

Studies meeting inclusion criteria focused primarily on patients with ABD, with outcomes focused on rates of assault and ABDs, restraint use, staff perception and weapon detection. Several studies focused on more than one intervention. Three^3^,^7^,^26^,^27^ implemented environmental strategies; three^3^,^7^,^27^ reported on policy interventions, and seven^3^,^7^,^26^,^28^–^31^ reported results of changes to practice. All studies were rated as being of weak quality (Table 2).

### Narrative Synthesis

Casteel et al. described an analytic cohort to investigate how the California (CA) Hospital Safety and Security Act (CHSSA) of 1995 affected violent events against hospital employees in CA EDs three years pre-enactment and six years post-enactment.^27^ The CHSSA required prevention and response interventions plans including environmental, security, policies and surveillance of violent events. New Jersey (NJ) EDs were used as temporal controls. Ninety-five CA and 46 NJ hospitals participated. Occupational Safety and Health Administration (OSHA) data were used to record violent injuries towards employees (physical contact and/or verbal assault) per 100,000 employee hours per year. Violent-event data were identified within OSHA logs, employers’ reports and supporting documentation. Violent assaults abstracted were mostly physical (90%). The requirement to report only events producing employee injury necessitating absenteeism or more than first-aid is likely to have minimised event detection. Subsequently, very few events were recorded in each group.

Results indicated a decrease in assaults per 100,000 employee hours per year after policy enactment in CA EDs (0.68 to 0.60), while there was an increase in NJ EDs over the same period (0.55 to 0.62). Several factors may have produced underestimation of results including unrecorded, underreported and missing violence data. Lack of differentiation between hospital staff and contractors may have confounded results since contractor hours were not recorded. Differences in the sociodemographics of the populations may have impacted violent-event frequencies. Additionally, lack of baseline data before CHSSA introduction precluded analysis of change in legislation compliance. It is of concern that there were highly unequal participation rates for CA (93%) and NJ hospitals (65%). In addition, control sites may have refused participation if they perceived their management of violence to be poor. The study was strengthened by the use of mandated documents, a large, diverse sample, and a sampling strategy that included rural and urban trauma facilities, and general acute care <300 beds and ≥300 beds. Additionally, there was consideration of confounders in the effect-modifiers between those with and without missing OSHA data. While the policy may have led to the observed difference in assault rates, the long-term effect of such policy change regarding maintained compliance and impact needs to be further assessed.

Cowling et al. presented a retrospective audit of behavioural assessment room (BAR) use within a single ED, together with an interrupted time series to evaluate the BAR as an ABD management strategy within the ED, assessed by staff survey.^7^ The intervention involved the creation of a specialised room enabling ABD management away from the main ED area, and the introduction of associated policy. The audit was a 12-month retrospective evaluation of the BAR use by ED patients with ABD, five months post-intervention introduction. For the post-intervention questionnaire, responses were obtained from 80/110 possible ED clinical, non-clinical and security staff (72.7% response rate). A pre-intervention questionnaire was undertaken two years prior to this study. The post-intervention questionnaire was completed 10 months post-introduction of the BAR and associated policy. The study may have been limited by recall bias and the failure to use a reliable, validated tool to assess perceptions towards ABDs. Despite the high survey-response rate (73%) a test-retest approach could not be undertaken due to staff turnover. Selection bias and confounding may have impacted the study with no assessment of non-BAR ABD population characteristics, nor the comparative characteristics for questionnaire responders and non-responders. In addition, the audit’s length of one year may have impacted on the ability of the study to investigate potential trends over time. The fact that all BAR ABD patients were audited would have minimised selection bias.

Emde et al. undertook a retrospective interrupted time series to evaluate whether increasing safety of seclusion rooms, together with staff education regarding restraint use and improved restraint documentation, affected ED restraint use.^26^ Thirty-six medical charts were audited prior to intervention (March–May 2000), which commenced June 2000, and 15 charts post-intervention (October–December 2001). Participants were ED staff, mental health aides/sitters, and patients who were restrained. Outcomes included percentages of accurate documentation recorded, as well as the number of staff injuries, restrained patients, and participants undertaking training. Emde et al. found that fewer restraints were used (20 per month to 7) post-intervention with no increase in injury to staff. Notable limitations include the following: limited detail presented in methods and results; no discussion of participant characteristics; possible attrition bias affecting injury data. The interventions may have acted as confounders as each may have individually both increased or decreased the number of violent events. Furthermore, it was unclear what percentage of staff completed training prior to the post-intervention audit that began prior to the stated 100% staff completion mentioned in December. The reduction in charts audited between the two periods could be due to reduced seclusion and/or restraint; however, this point was not made. Particular aspects of the intervention were cited as producing an “inability to use restraints on the new beds,” and the requirement to use beds in other rooms may have contributed to the overall restraint rate and use of the seclusion room affecting the validity of the findings presented. Further concern regarding validity of results arises from the lack of statistical analysis description or confidence intervals for the outcome measures.

Rankins et al. undertook a retrospective interrupted time series to assess the effectiveness of a security system with metal detectors in a single urban ED.^30^ Records were retrieved for 29 months pre-implementation and 25 months post-implementation covering 1992–1996. Outcome measures included rates of assaults per 10,000 ED patients treated and the percentage of weapons confiscated. Although reported assaults did not change significantly, the rate of weapon confiscation was significantly reduced at post-implementation compared to the period before the introduction of the security system, with the greatest difference observed for the patient treatment area (pre: 92%; post: 42%, p<0.001). That is, there was a higher rate of detection prior to attending the treatment area. The study was weakened by the use of one data extractor; the use of retrospective data, which limited the ability to estimate non-documentation; the inability to assess how many weapons were missed by the security system; and the inability to differentiate whether more people were bearing weapons or whether more weapons were being detected. Overall, this study demonstrated that a security system may assist in weapon detection and confiscation, but does not provide evidence for a reduction in assault rates.

Gillespie et al. reported a prospective, non-randomised controlled trial involving three intervention and three comparison sites matched by ED type (Level 1, urban tertiary care, community)^3^. Allocation to intervention was randomly assigned, and participants were eligible if they worked >20 hours a week and provided direct patient care. Intervention sites received a workplace violence intervention comprising unspecified environmental changes, policies, procedures and education over three months in 2010. Outcomes were assessed during the nine months before and nine months after the intervention using a baseline demographic survey, a monthly survey (number of assaults and physical threats in preceding month), and a violent-event survey recording details of the perpetrator. Results indicated a decrease in assault rates for intervention groups and control sites, but no differences between controls and intervention sites after accounting for pre-intervention differences. Although there was no mention of interaction effects (time X allocation), post-hoc analyses of individual intervention sites were reported. Between-group differences in change scores (from baseline) would have been a more appropriate method of analysing assault rates and threats. The study may have been weakened by recall bias, reporting bias due to being increasingly aware of violence, survey fatigue, and the inability to randomise participants to treatment or control. Additionally, there was a preponderance of female and nurses among participants. The study was strengthened by stratification of the intervention and control groups according to ED type.

McMahon et al. performed a mixed-method study involving an interrupted time series, pre-intervention interviews, and post-intervention staff survey.^29^ The intervention involved new restraint documentation, assessment of security personnel deployment, de-escalation/self-defence training and an adoption of a “zero-tolerance policy.” Data were collected over three years and included average restraints per month, diagnosis and patient disposition. Staff were interviewed about level of satisfaction with restraint documentation and attitudes with restraint interventions, and were also surveyed on demographics, use of restraint, assaults on witnesses and themselves, the response and attitude to assaults. Average monthly restraint decreased (from 37 to 21), as did the restraint duration (2.3 hours to 1.9 hours) following intervention implementation. Although strengthened by the multiple methods for assessing reduction in restraint and attitudes, the study was limited by missing data that increased potential attrition bias, as well as the failure to record restraint as a percentage of ABD episodes. It is unclear whether seasonal differences accounted for changes in the need for restraint. Finally, insufficient description of methods makes reproducibility and interpretation of the study difficult, particularly for data extraction and analysis, and consideration of bias and confounding.

Griffey et al. performed a prospective cohort study examining the effect of a forcing function within a computerized ED order-entry system on the timeliness of renewal of restraint orders.^31^ The study period was between July 2003–December 2004 and consisted of six months baseline, six months of a computerised forcing function that allowed acknowledgment or renewal of the restraint without consequence (hereafter, “soft stop”), and a subsequent six months wherein the computerized forcing function that required addressing before enabling access to the ED information system (hereafter, “forced function”). The reminder and lockout system were tracked to the physician managing the restraint of a particular patient. The primary outcome was median time to restraint-order renewal before and after successive implementation of the forcing function. Secondary outcomes included mean number of restraint orders per patient, mean number of renewal orders per hour a patient was restrained, and median patients spent in restraints, all with comparisons of variability in these measurements. A non-significant reduction in time in restraint was reported, as was an improvement in restraint reordering (mean number of orders per hour: baseline 0.08; soft stop 0.23; forced function 0.89. Mean number of restraint orders per patient: baseline 1.46; soft stop 1.89; forced function 2.34. Mean renewal of orders: baseline, 228 minutes; soft stop, 149 minutes; forced function, 140 minutes) and variability in practice. There are several study limitations. The maximum number of restraint orders per person was truncated to seven leading to an underestimation due to a ceiling effect. Further, orders for restraints included those for physical restraint, seclusion, and sitter/observers but not “chemical restraint.” Discontinuation orders were not specifically assessed, limiting the impact the intervention had on the practice of allowing orders to expire rather than behaviourally indicated discontinuation. Strengths of the study include the use of a computerised system allowing easy data acquisition; the selected targeting of doctors who ordered particular restraint allowing for accountability of staff; the six-month interval may have provided sufficient time for adjustment to intervention iterations; and the generalisability of the program given the only requirements are computer-based systems and a tracking system. Given that all doctors who issued restraint orders were involved the potential for selection bias was reduced.

Cailhol et al. undertook an interrupted time series with data collected five months pre- and post-multimodal intervention involving education, staff dialogue in meetings and journal club, medical presence during restraint interventions, and debriefing following restraint.^28^ Data were collected by clinician survey, and results indicated a reduction in ABDs compared to pre-intervention. The study lacked a temporal control, and there was no blinding of clinicians receiving the intervention and making decisions about restraint use. Additionally, it was a single-centre study involving a psychiatric emergency hospital that may limit any generalisability of the study findings. Of note, the main outcome measure was percentage of violent patients (as a function of total presentations), rather than rate of violent behaviour. This is critical given that more than one assault may occur by the same individual.

## DISCUSSION

### Summary of main results

ABDs within EDs are of great concern given their potential negative impact on wellbeing, retention, safety and performance of staff, as well as the impact on patient care and safety. This systematic review assessed the efficacy of non-pharmacological interventions for managing ED ABDs. Using our comprehensive search criteria, the number of interventions we identified that were specific to the ED were limited. Eight studies met pre-set criteria for inclusion with several incorporating multiple intervention components involving changes to environment, policy and practice rather than assessment of single interventions. Heterogeneity of study designs and outcome measures limited analysis to narrative synthesis. Alarmingly, despite searching a publication period spanning three decades, no study provided a level of evidence sufficient to warrant recommendation for any specific intervention.

### Quality of the Evidence

All studies included in this review were rated as having weak quality. It is therefore inappropriate to make recommendations to uptake strategies to limit ABDs. Included studies were hampered by multiple factors: Although study designs were primarily interrupted time series and thus rated as having moderate quality for this criterion, several were subject to selection bias, most had problems with blinding and weak data collection methods, and studies were uniformly weak in terms of being affected by participant withdrawal or dropout. The degree to which studies were affected by confounders was variable. Others have noted the lack of quality evidence in this field. Nelstrop et al. reviewed the literature from 1985–2002 and found no evidence from comparative studies for or against the use of physical restraint and/or seclusion in the management of short-term ABDs within the adult psychiatric in-patient setting.^18^ Similarly, Anderson et al. identified studies reporting management approaches of violence directed against emergency nurses (1986–2007); studies were of poor quality and were largely focused on defining the phenomenon instead of developing effective management methods.^10^

### Applicability of evidence

The definition and description of interventions can profoundly affect interpretation of evidence and the way in which components of interventions are understood to have an effect on outcomes. In some cases (e.g., Emde^26^) interventions were poorly described, and for others (e.g., Rankins^30^) there was poor compliance with expected conventions for reporting. While all studies included were relevant to the ED setting, the specific context of interventions may have been affected by the small-scale nature of the studies. Patient demographics vary from ED to ED and while certain demographic profiles may provide a strong impetus for change in practice, assessments of efficacy for such changes must consider generalisability to other EDs.

Interventions described by studies reviewed here frequently modified multiple variables, possibly reflecting a real-world approach to the problem. Complex interventions with multiple components make it difficult to isolate and neutralize the influence of confounders as well as the relative influence of each intervention. Despite problems inherent to multifaceted interventions, it is pertinent that Casteel et al.’s^27^ multifaceted prevention and response intervention (environmental, security, policies and surveillance) significantly reduced assault rates. Although of “weak” quality, this study adopted an approach whereby the exact prevention and response interventions implemented were not uniform across hospitals. Instead, each hospital identified and implemented the interventions deemed most relevant and feasible for each site.

It is clear that further studies are required to robustly evaluate the efficacy of management strategies in multi-site and multi-disciplinary studies to provide better evidence for interventions aimed at reducing the occurrence of ABDs within EDs.

Efficacy studies have been hampered by a lack of unifying definition for the phenomena under investigation with some adopting a broad umbrella term such as ABD, and others focusing on specific forms of violence, such as physical assault. Conceivably, in the search for hard outcome measures with unambiguous definition, physical assault has become the default outcome measure. Given the difficulties associated with documenting verbal assault, reliance on physical assault as the endpoint will under-represent the true prevalence of what most clinicians experience as assault. Establishing and supporting routine surveillance across all settings that truly reflects the incidence of ABD is the first step in moving towards protection of healthcare workers.

Studies in this field have been hampered by a lack of standardisation for assessing efficacy of methods aimed at reducing ABD. Clearly, there is no accepted standard rate of measuring assault, for example, with some using the sample population denominator, others expressing assault as a rate (e.g., per 10,000 patients), and yet others focusing on proportion of perpetrators rather than events. The lack of validated measures for some psychosocial outcomes is also problematic. While validated tools with sound psychometric properties exist for the assessment of clinician and patient attitudes to the management of violence,^32^ it was not uncommon for studies included to adopt purpose-designed tools, the characteristics of which have not been tested rigorously. Overall, this lack of standardisation and limited use of rigorous tools limits the quality and comparability of research in this field, and is an important consideration when designing future studies.

### The Way Forward

The present review has revealed an unambiguous gap in research. While this should provide impetus for directing next steps, as others have noted,^33^ a lack of unified research effort in this field remains despite previous calls for solutions.^10^,^34^ The shift toward building a sound evidence base in the non-pharmacological management of ABDs in EDs that is unified requires a coordinated approach, with cooperation across multiple sites. Our review revealed that the existing evidence base typically comprises single-site studies, with multiple different techniques and modalities employed. Whilst it is important for EDs to respond to the local environment, resources, staff and population, and develop interventions accordingly, the field would benefit from a greater emphasis on collaborative, multi-centre, suitably funded studies that may afford superior study designs and execution.

Despite weak, preliminary evidence for efficacy of specialised rooms such as BARs, architectural changes to manage ABDs are becoming common in EDs. These spaces may be perceived by hospital managers as having a potential preventive benefit, and given the pressing need to maintain staff safety, managers may not have the luxury of awaiting a sound body of evidence. Nonetheless, this begs the question: Do specialised rooms assist in ED ABD management, and if so what format is best? Given the variable nature of EDs, it is unlikely that a one-size-fits-all approach will be suitable. Going forward, there is a definite need to balance flexible, tailorable interventions with a unified approach that facilitates larger scale, multi-site studies, and respects all local legal requirements.

There is little evidence that the ABD management strategies reviewed here are effective. In the clinical practice of employing any restrictive interventions, respect for human rights should be the paramount guiding principle; clinicians should employ the least restrictive means to provide a safe environment for both staff and patient. Guidelines^35^–^38^ support a graded response from verbal de-escalation, to pharmacological means, with manual/mechanical restraint and seclusion the last resort. Additionally, clinicians should refer to existing legal frameworks as a reference point within which to work.

## STRENGTHS AND LIMITATIONS OF THIS REVIEW

The search was limited to the English language. This may have biased against articles written in languages other than English, which may have prevented identification of relevant interventions for managing ABD. The same is true for the restriction to published literature and not to include grey-literature databases.

Our inclusive search terms produced a broad array of study designs and outcome measures. Whilst this resultant heterogeneity prevented meta-analysis and rendered narrative synthesis necessary, the inclusive search is a study strength as more studies would likely be identified. Other strengths of this study were the application of a critical appraisal tool by two independent abstractors to consider quality of evidence. The lack of blinding of these data abstractors against study aims, however, is a study limitation. The study was also limited by the inability to source three full-text papers deemed potentially relevant. Since EDs are of significant heterogeneity, the studies included in this review may not be representative of all EDs’ patients and staffs across regions.

## CONCLUSION

In the absence of well-controlled studies, no recommendations can be made about the efficacy of non-pharmacological strategies to manage ABDs within EDs. While ABD management interventions show a level of innovation, and may still be practical and safe, some are highly resource intensive. Further, more rigorous testing of efficacy for interventions designed to manage ABDs in EDs is essential. Continued practice of these strategies should be undertaken only in the context of ongoing evaluations of both efficacy and safety. The impetus for effective, evidence-based ABD management within the ED is escalating. The time is now for further research that is robust, multi-site, widely applicable or flexible, large in sample size, over significant periods and involving qualitative and quantitative evidence.

## Supplementary Information









## Figures and Tables

**Figure:**
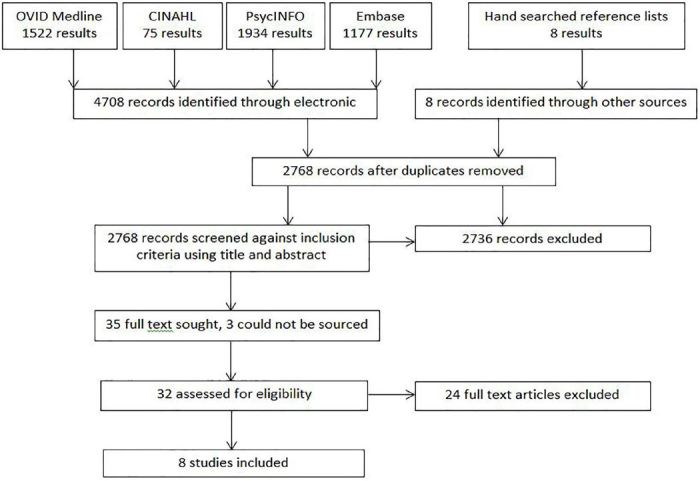
Systematic search results in a review of the efficacy of strategies for managing acute behavioural disturbances in the emergency department.

**Table 1:** Summary of data extracted from studies included in a review of strategies to deal with acute behavioural disturbances by patients in the emergency department.

Primary Author (Year)	Setting, Country	Study Design & Duration	Participants (Type, N, selection, characteristics)	Interventions or Exposures	Data collection methods	Main Outcome Measures	Main Findings	Possible Confounders and biases
Gillespie (2014)^3^	The settings included 2 level I trauma centers, 2 urban tertiary care EDs, and 2 community-based suburban EDs	Controlled trial (quasi experimental). 18 month study period (9m pre, 9m post), Sites matched by type and then randomly assigned as intervention or comparison sites.	209/213 eligible participants. 71% female; 56% nurses.	environmental changes, policies and procedures, and education and training	3 researcher devised surveys: baseline demographic survey; monthly survey; and Violent Event Survey.	Mean rate of staff reported assaults and physical threats	Rate of assaults decreased significantly over time for both intervention groups and controls No significant differences in assault or physical threat rates based on gender, occupation, and ED type.	Unclear whether there was a direct comparison of change in assault rates between controls and intervention group. Inappropriate use of post-hoc testing. Individual participants not randomised. Lack of clarity regarding uniformity of intervention (sites were given key elements of policy only).
Casteel (2009)^27^	California (CA)and New Jersey (NJ) emergency departments (EDs) and Psychiatric Units. USA	Analytic cohort; Duration: 9 years (pre: 1993–1995; post: 1996–2001).	95 EDs in CA; 46 in NJ (control). No baseline data; Selection based on locality; uneven participation rates (93%, 65%); Whole of hospital data only	Enactment of the California Hospital Safety and Security Act	Assaults: Occupational Safety and Health Administration Logs, Employer Reports, hospital incident reports, supervisor reports security logs Employee hour data: Electronic Records	Assault rates per 100,000 employee hours per year.	Assault rates decreased 48% in CA post-enactment, compared with New Jersey (rate ratio = 0.52, 95% CI: 0.31, 0.90).	Uneven participation rates; Hospital staff and contract workers not differentiated in OSHA data. No blinding; Missing employee hour data mainly for CA hospitals; Higher ratio of severe events reported. Likely under-reporting of incidents.
Cowling (2007)^7^	ED of Major metropolitan teaching hospital, Australia.	(1) interrupted times series; (2) 12m audit (2003)	(1) Audit: n=117 patients managed in behavioural assessment room (BAR): age range = 19.7–61.7 years; 76 male; 38 female; 3 no gender specified) (2) Cohort: 80/110 ED staff; staff self-referred, high attrition resulting in inclusion of staff at post-intervention that were not present at pre-intervention.	Use of a specialized behavioural assessment room (BAR); BAR policy, staff education; and team response.	Retrospective audit using pre-defined form; researcher devised survey	Duration in BAR; restraint method; patient clinical and demographics characteristics Staff type; incidence and frequency of verbal or physical abuse before and after intervention; perceived safety; policy awareness, perceived effectiveness of policy; violence-related absenteeism; effects of violence; perceived impact on care, response times; whether the intervention had supported	Median duration of BAR use: 20 min; 65.8% patients restrained; 23.3% chemical restraint alone, 28.5% Mechanically restrained alone; 29.8%, both Mechanical and chemical 58% intoxicated. Questionnaire Results: 44% Affected personally by violence; 14.9% required time off; 87.5%; verbally assaulted; 52.1% physically assaulted; 98.5% believed that the BAR created a safer environment; 86.5% of all respondents reported feeling safe; 74.5%; reported the BAR policy improved management of patients; 63.6% noted more timely response to patient management.	Recall bias; No reliable and validated tools, potential for selection bias. The percentage of missing data would affect results robustness and lower estimated results. The questionnaire post-intervention was modified from that performed 2 years ago (pre-intervention) complicating direct comparison. No blinding reported.
Cailhol (2007)^28^	Emergency Psychiatric Department, of a single hospital, Geneva, Switzerland.	interrupted times series, 5 month pre, 5 months post)	478 patients attending during 10 month study period. Pre: 254 Post: 224)	Education focused on restraint and violent behavior Dialog between staff through meetings, including a journal club Medical presence during all security interventions for restraint Debriefing after restraint use	Ad hoc questionnaire of patient behaviour completed by clinicians	% Violent patients (as a function of total presentations)	A significant reduction in VB (was found before and after the intervention (17% to 7%). No significant differences for sex, age and diagnosis between the two periods between patients with VB and patients without VB.	Absence of a temporal control. No blinding: Clinicians making decisions about restraint were also those collecting data.
McMahon (2003)^29^	Urban level 1 trauma centre Boston Medical Centre (BMC)	interrupted times series ; (Pre: Jan–July 2000; Post: 2001, dates not specified) Interviews: Pre: Sept–Oct 2000; Post: 2001, dates not specified. Post-intervention Survey April 2001.	62 ED nurses from trauma, paediatric and cardiac areas. 84% Female, 50% >41 yrs; Self selected. Characteristics of patients participants not documented.	Modified restraint documentation tool + training	Audit data; interviews; surveys	Restraint episodes per months; qualitative feedback from staff	Restraint episodes reduced from 37/month to 21/month; Duration of retrain decreased from 2.3 hrs to 1.9 hrs; Staff reported heightened sense of safety.	No inferential analyses on final outcomes. Unclear what the post-intervention dates were. No blinding reported.
Rankins (1999)^30^	A single urban ED, California, USA	Retrospective audit of interrupted times series 54 months, 1992–1996	264,970 patient attendances (155,976 pre-intervention; 108,994 post-intervention). No characteristics provided. Participant selected based on period of observation and security records of outcomes.	Implementation of a security system incorporating metal detectors	Security records	Rate of weapons confiscated per 10,000 ED pts; Number of assaults per 10,000 ED patients	No change in reported assaults per 10, 000 persons. A significant greater number of weapons (per 10,000 persons) were confiscated post-intervention compared to baseline (24 vs 40). The percentage of weapons confiscated in the patient are decreased significantly over time (92% to 42%).	Excluded verbal assaults; and patients that required restraint. Chart abstractors and data assessors not blinded. Retrospective data subject to bias due to non-documentation. Results may have been attributable to more people carrying weapons over the study period resulting in increased rates of detection. The number of violent events was rare due to the definition of assault used.
Emde (2002)^26^	ED of a single level III community hospital (USA)	Retrospective audit of interrupted times series. Pre-intervention: Mar–May; Post intervention: Oct–Dec 2001	51 restrained patients, characteristics not provided; ED staff: number and details not specified. No baseline data for staff or patient characteristic provided; 100% of staff exposed to intervention	Safety modifications to seclusion room; Restraint and de-escalation education; Restraint form with monthly review of charts Psychiatric staff used as sitters for seclusion observation	Purpose designed forms; unclear how injuries documented; attendance records	Restraint forms; #injuries to staff; #restrained patients; # staff trained	Fewer restraint used (20 per month to 7) post-intervention with no increase in injury to staff. No tests of significance. Decrease in damage to seclusion room.	Limited methodological information provided Participant characteristics not provided. Staff training completed by 100% staff by Dec 2001, but post-intervention audit was Oct–Dec 2001 thus ongoing staff training may have affected results. Modification of outcome measures between the pre- and post-intervention limits comparisons Attrition bias likely as incomplete data presented. Data for injury to staff not provided. The “inability to use restraints on the new beds” and the requirement to use beds in other rooms may have impacted on the amount of restraint use confounding results. Inferential statistics not provided No blinding reported.
Griffey (2009)^31^	Academic, urban, adult-only, Level I ED (USA)	Prospective Cohort July 2003 and December 2004. 3 successive 6-month blocks	All doctors that had restraint orders for patients during the study period (including physical restraints, seclusion, and sitter/observers). Patients: 139 patient visits across the 3 study intervals included restraint renewal orders, with a total of 261 renewal orders in all.	(1) baseline; (2) computerised forcing function, allowing acknowledgement or renewal of restraint orders without consequence; (3) computerized forcing function with a requirement of addressing before enabling access to the ED information system.	Data source: Charts where restraint orders were given within the study period. The use of a query search using the computerized order entry system database included: patient age and sex; indication for restraint, restraint type ordered, and patient disposition.	Time to order renewal, number of restraint orders, renewal orders per hour in restraints, and time in restraints	Median time to order renewal decreased in periods 1 and 2 versus baseline by 64 and 56 minutes. Mean number of restraint orders in periods 2 and 3 significantly increased vs those in baseline (1.46 to 1.89 to 2.34). Mean renewal orders per hour in restraint significantly increased in period 2 versus baseline and 2, from 0.08 to 0.23 to 0.89. Non significant decreases in median time spent in restraints observed in periods B and C versus baseline of 45 and 105 min.	Underestimation of the number of restraint orders for an individual as the orders captured were truncated to a maximum of 7. Assessment included physical restraint only Orders for discontinuation were not specifically addressed and No quantification of orders discontinued or allowed to expire. No blinding undertaken.

**Table 2:** Quality-of-evidence rating based on the Effective Public Health Practice Project (EPHPP) Quality Assessment Tool for Quantitative Studies.

First Author (year)	Selection bias	Study design	Confounders	Blinding	Data collection method	Withdrawals and dropouts	Global rating
Cailhol (2007)^28^	Weak	Moderate	Moderate	Moderate	Weak	Weak	Weak
Casteel (2009)^27^	Weak	Moderate	Moderate	Weak	Weak	Weak	Weak
Cowling (2007)^7^	Weak	Moderate	Weak	Weak	Weak	Weak	Weak
Gillespie (2014)^3^	Moderate	Strong	Strong	Weak	Weak	Weak	Weak
McMahon(2003)^29^	Weak	Weak	Weak	Weak	Weak	Weak	Weak
Rankins (1999)^30^	Moderate	Moderate	Moderate	Weak	Weak	Weak	Weak
Emde (2002)^26^	Weak	Moderate	Weak	Weak	Weak	Weak	Weak
Griffey (2009)^31^	Moderate	Moderate	Moderate	Weak	Moderate	Weak	Weak
