# Supplementary material for: Managing Acute Behavioural Disturbances in the Emergency Department Using the Environment, Policies and Practices: A Systematic Review
Source: West J Emerg Med. 2017 May 15;18(4):647–61. doi: 10.5811/westjem.2017.4.33411 (PMC5468071; doi:10.5811/westjem.2017.4.33411)
Supplement: Supplementary file 1 [file wjem-18-647-s001.docx]

APPENDIX A: Search undertaken using OVID Medline

| 1. (emergency department* or ED* or "A&E" or emergency room*).m_titl. |  |
| --- | --- |

| 2. *Emergency Medical Services/ |  |
| --- | --- |

| 3. emergency medicine.mp. or Emergency Medicine/ |  |
| --- | --- |

| 4. 1 or 2 or 3 |  |
| --- | --- |

| 5. (aggression or difficult or demand* or disrupt* or assault* or threat* or antagonistic or hostile or combat* or confront* or argument or behavio* or challeng*).ti. |  |
| --- | --- |

| 6. *exposure to violence/ or *workplace violence/ |  |
| --- | --- |

| 7. violence/ or workplace violence/ |  |
| --- | --- |

| 8. "violen*".m_titl. |  |
| --- | --- |

| 9. *Agonistic Behavior/ |  |
| --- | --- |

| 10. Patient Self-Determination Act/ |  |
| --- | --- |

| 11. 5 or 6 or 7 or 8 or 9 or 10 |  |
| --- | --- |

| 12. patient isolation.mp. or Patient Isolation/ |  |
| --- | --- |

| 13. *Immobilization/ |  |
| --- | --- |

| 14. *health facility environment/ or patients' rooms/ |  |
| --- | --- |

| 15. confined spaces/ |  |
| --- | --- |

| 16. *Environment Design/ |  |
| --- | --- |

| 17. (isolat* or confine* or hold or restrain* or room* or sensory*).tw. |  |
| --- | --- |

| 18. *risk reduction behavior/ |  |
| --- | --- |

| 19. *Risk Assessment/ |  |
| --- | --- |

| 20. organizational policy.mp. or Organizational Policy/ |  |
| --- | --- |

| 21. (policy or management or intervention).tw. |  |
| --- | --- |

| 22. 12 or 13 or 14 or 15 or 16 or 17 or 18 or 19 or 20 or 21 |  |
| --- | --- |

| 23. 4 and 11 and 22 |  |
| --- | --- |

| 24. limit 23 to (english language and yr="1985-Current") |  |
| --- | --- |
