# Supplementary material for: Managing Acute Behavioural Disturbances in the Emergency Department Using the Environment, Policies and Practices: A Systematic Review
Source: West J Emerg Med. 2017 May 15;18(4):647–61. doi: 10.5811/westjem.2017.4.33411 (PMC5468071; doi:10.5811/westjem.2017.4.33411)
Supplement: Supplementary file 2 [file wjem-18-647-s002.docx]

**APPENDIX B: Search undertaken using PsycInfo**

| 1. emergency department.tw. |  |
| --- | --- |

| 2. emergency room.tw. |  |
| --- | --- |

| 3. emergency medicine.tw. |  |
| --- | --- |

| 4. (emergency department* or ED* or "A&E" or emergency room*).m_titl. |  |
| --- | --- |

| 5. 1 or 2 or 3 or 4 |  |
| --- | --- |

| 6. *Aggressive Behavior/ |  |
| --- | --- |

| 7. *behavior problems/ |  |
| --- | --- |

| 8. violence/ or antisocial behavior/ or conflict/ or workplace violence/ or exposure to violence/ |  |
| --- | --- |

| 9. (aggression or difficult or demand* or disrupt* or assault* or threat* or antagonistic or hostile or combat* or confront* or argument or behavio* or challeng*).m_titl. |  |
| --- | --- |

| 10. "violen*".m_titl. |  |
| --- | --- |

| 11. Agonistic Behavior.mp. |  |
| --- | --- |

| 12. 6 or 7 or 8 or 9 or 10 or 11 |  |
| --- | --- |

| 13. *patient seclusion/ or exp patient violence/ |  |
| --- | --- |

| 14. *physical restraint/ |  |
| --- | --- |

| 15. *hospital environment/ |  |
| --- | --- |

| 16. patients' rooms.mp. |  |
| --- | --- |

| 17. (isolat* or confine* or hold or restrain* or room* or sensory*).tw. |  |
| --- | --- |

| 18. *risk assessment/ |  |
| --- | --- |

| 19. exp health care policy/ |  |
| --- | --- |

| 20. organizational policy.mp. |  |
| --- | --- |

| 21. (policy or management or intervention).tw. |  |
| --- | --- |

| 22. 13 or 14 or 15 or 16 or 17 or 18 or 19 or 20 or 21 |  |
| --- | --- |

| 23. 5 and 12 and 22 |  |
| --- | --- |

| 24. limit 23 to (english language and yr="1985-Current") |  |
| --- | --- |
