# Supplementary material for: Managing Acute Behavioural Disturbances in the Emergency Department Using the Environment, Policies and Practices: A Systematic Review
Source: West J Emerg Med. 2017 May 15;18(4):647–61. doi: 10.5811/westjem.2017.4.33411 (PMC5468071; doi:10.5811/westjem.2017.4.33411)
Supplement: Supplementary file 3 [file wjem-18-647-s003.docx]

APPENDIX C: Search undertaken using CINAHL Plus

| S39 | S6 AND S23 AND S37 | Limiters - Published Date: 19850101-20151231  Narrow by Language0: - English  Search modes - Boolean/Phrase | 70 | [Edit](javascript:__doPostBack('ctl00$ctl00$MainContentArea$MainContentArea$editControl$printHistory$HistoryRepeater$ctl00$linkEditSearch','')) S39 |
| --- | --- | --- | --- | --- |
| S38 | S6 AND S23 AND S37 | Search modes - Boolean/Phrase | 78 | [Edit](javascript:__doPostBack('ctl00$ctl00$MainContentArea$MainContentArea$editControl$printHistory$HistoryRepeater$ctl01$linkEditSearch','')) S38 |
| S37 | S24 OR S25 OR S26 OR S27 OR S28 OR S29 OR S30 OR S31 OR S32 OR S33 OR S34 OR S35 OR S36 | Search modes - Boolean/Phrase | 751,756 | [Edit](javascript:__doPostBack('ctl00$ctl00$MainContentArea$MainContentArea$editControl$printHistory$HistoryRepeater$ctl02$linkEditSearch','')) S37 |
| S36 | (polic* or management or intervention) | Search modes - Boolean/Phrase | 606,722 | [Edit](javascript:__doPostBack('ctl00$ctl00$MainContentArea$MainContentArea$editControl$printHistory$HistoryRepeater$ctl03$linkEditSearch','')) S36 |
| S35 | Organizational Polic* | Search modes - Boolean/Phrase | 9,743 | [Edit](javascript:__doPostBack('ctl00$ctl00$MainContentArea$MainContentArea$editControl$printHistory$HistoryRepeater$ctl04$linkEditSearch','')) S35 |
| S34 | (MM "Organizational Policies") | Search modes - Boolean/Phrase | 3,077 | [Edit](javascript:__doPostBack('ctl00$ctl00$MainContentArea$MainContentArea$editControl$printHistory$HistoryRepeater$ctl05$linkEditSearch','')) S34 |
| S33 | "*Risk Assessment" | Search modes - Boolean/Phrase | 62,398 | [Edit](javascript:__doPostBack('ctl00$ctl00$MainContentArea$MainContentArea$editControl$printHistory$HistoryRepeater$ctl06$linkEditSearch','')) S33 |
| S32 | (MH "Risk Management (Iowa NIC)+") | Search modes - Boolean/Phrase | 30 | [Edit](javascript:__doPostBack('ctl00$ctl00$MainContentArea$MainContentArea$editControl$printHistory$HistoryRepeater$ctl07$linkEditSearch','')) S32 |
| S31 | TI (isolat* or confine* or hold or restrain* or room* or sensory*) | Search modes - Boolean/Phrase | 28,737 | [Edit](javascript:__doPostBack('ctl00$ctl00$MainContentArea$MainContentArea$editControl$printHistory$HistoryRepeater$ctl08$linkEditSearch','')) S31 |
| S30 | (isolat* or confine* or hold or restrain* or room* or sensory*) | Search modes - Boolean/Phrase | 114,121 | [Edit](javascript:__doPostBack('ctl00$ctl00$MainContentArea$MainContentArea$editControl$printHistory$HistoryRepeater$ctl09$linkEditSearch','')) S30 |
| S29 | *Environment Design | Search modes - Boolean/Phrase | 126 | [Edit](javascript:__doPostBack('ctl00$ctl00$MainContentArea$MainContentArea$editControl$printHistory$HistoryRepeater$ctl10$linkEditSearch','')) S29 |
| S28 | confined spaces | Search modes - Boolean/Phrase | 399 | [Edit](javascript:__doPostBack('ctl00$ctl00$MainContentArea$MainContentArea$editControl$printHistory$HistoryRepeater$ctl11$linkEditSearch','')) S28 |
| S27 | (MH "Patient Seclusion/PF") | Search modes - Boolean/Phrase | 12 | [Edit](javascript:__doPostBack('ctl00$ctl00$MainContentArea$MainContentArea$editControl$printHistory$HistoryRepeater$ctl12$linkEditSearch','')) S27 |
| S26 | Patient Isolation | Search modes - Boolean/Phrase | 2,020 | [Edit](javascript:__doPostBack('ctl00$ctl00$MainContentArea$MainContentArea$editControl$printHistory$HistoryRepeater$ctl13$linkEditSearch','')) S26 |
| S25 | patients' rooms | Search modes - Boolean/Phrase | 1,108 | [Edit](javascript:__doPostBack('ctl00$ctl00$MainContentArea$MainContentArea$editControl$printHistory$HistoryRepeater$ctl14$linkEditSearch','')) S25 |
| S24 | "Immobilization" | Search modes - Boolean/Phrase | 4,090 | [Edit](javascript:__doPostBack('ctl00$ctl00$MainContentArea$MainContentArea$editControl$printHistory$HistoryRepeater$ctl15$linkEditSearch','')) S24 |
| S23 | S7 OR S8 OR S9 OR S10 OR S11 OR S12 OR S13 OR S14 OR S15 OR S16 OR S17 OR S18 OR S19 OR S20 OR S21 OR S22 | Search modes - Boolean/Phrase | 124,725 | [Edit](javascript:__doPostBack('ctl00$ctl00$MainContentArea$MainContentArea$editControl$printHistory$HistoryRepeater$ctl16$linkEditSearch','')) S23 |
| S22 | TI "behavio*" | Search modes - Boolean/Phrase | 56,974 | [Edit](javascript:__doPostBack('ctl00$ctl00$MainContentArea$MainContentArea$editControl$printHistory$HistoryRepeater$ctl17$linkEditSearch','')) S22 |
| S21 | TI "challeng*" | Search modes - Boolean/Phrase | 36,307 | [Edit](javascript:__doPostBack('ctl00$ctl00$MainContentArea$MainContentArea$editControl$printHistory$HistoryRepeater$ctl18$linkEditSearch','')) S21 |
| S20 | TI "argument*" | Search modes - Boolean/Phrase | 1,076 | [Edit](javascript:__doPostBack('ctl00$ctl00$MainContentArea$MainContentArea$editControl$printHistory$HistoryRepeater$ctl19$linkEditSearch','')) S20 |
| S19 | TI "confront*" | Search modes - Boolean/Phrase | 1,203 | [Edit](javascript:__doPostBack('ctl00$ctl00$MainContentArea$MainContentArea$editControl$printHistory$HistoryRepeater$ctl20$linkEditSearch','')) S19 |
| S18 | TI "combat*" | Search modes - Boolean/Phrase | 3,309 | [Edit](javascript:__doPostBack('ctl00$ctl00$MainContentArea$MainContentArea$editControl$printHistory$HistoryRepeater$ctl21$linkEditSearch','')) S18 |
| S17 | TI hostile | Search modes - Boolean/Phrase | 236 | [Edit](javascript:__doPostBack('ctl00$ctl00$MainContentArea$MainContentArea$editControl$printHistory$HistoryRepeater$ctl22$linkEditSearch','')) S17 |
| S16 | TI antagonistic | Search modes - Boolean/Phrase | 87 | [Edit](javascript:__doPostBack('ctl00$ctl00$MainContentArea$MainContentArea$editControl$printHistory$HistoryRepeater$ctl23$linkEditSearch','')) S16 |
| S15 | TI "assault*" | Search modes - Boolean/Phrase | 2,351 | [Edit](javascript:__doPostBack('ctl00$ctl00$MainContentArea$MainContentArea$editControl$printHistory$HistoryRepeater$ctl24$linkEditSearch','')) S15 |
| S14 | TI "disrupt*" | Search modes - Boolean/Phrase | 3,105 | [Edit](javascript:__doPostBack('ctl00$ctl00$MainContentArea$MainContentArea$editControl$printHistory$HistoryRepeater$ctl25$linkEditSearch','')) S14 |
| S13 | TI "demand*" | Search modes - Boolean/Phrase | 5,489 | [Edit](javascript:__doPostBack('ctl00$ctl00$MainContentArea$MainContentArea$editControl$printHistory$HistoryRepeater$ctl26$linkEditSearch','')) S13 |
| S12 | TI "difficult*" | Search modes - Boolean/Phrase | 8,742 | [Edit](javascript:__doPostBack('ctl00$ctl00$MainContentArea$MainContentArea$editControl$printHistory$HistoryRepeater$ctl27$linkEditSearch','')) S12 |
| S11 | TI "Aggress*" | Search modes - Boolean/Phrase | 5,991 | [Edit](javascript:__doPostBack('ctl00$ctl00$MainContentArea$MainContentArea$editControl$printHistory$HistoryRepeater$ctl28$linkEditSearch','')) S11 |
| S10 | (MM "Disruptive Behavior") | Search modes - Boolean/Phrase | 1,400 | [Edit](javascript:__doPostBack('ctl00$ctl00$MainContentArea$MainContentArea$editControl$printHistory$HistoryRepeater$ctl29$linkEditSearch','')) S10 |
| S9 | (MM "Verbal Abuse") | Search modes - Boolean/Phrase | 430 | [Edit](javascript:__doPostBack('ctl00$ctl00$MainContentArea$MainContentArea$editControl$printHistory$HistoryRepeater$ctl30$linkEditSearch','')) S9 |
| S8 | (MM "Patient Assault") | Search modes - Boolean/Phrase | 1,246 | [Edit](javascript:__doPostBack('ctl00$ctl00$MainContentArea$MainContentArea$editControl$printHistory$HistoryRepeater$ctl31$linkEditSearch','')) S8 |
| S7 | (MM "Workplace Violence") | Search modes - Boolean/Phrase | 2,241 | [Edit](javascript:__doPostBack('ctl00$ctl00$MainContentArea$MainContentArea$editControl$printHistory$HistoryRepeater$ctl32$linkEditSearch','')) S7 |
| S6 | S1 OR S2 OR S3 OR S4 OR S5 | Search modes - Boolean/Phrase | 9,162 | [Edit](javascript:__doPostBack('ctl00$ctl00$MainContentArea$MainContentArea$editControl$printHistory$HistoryRepeater$ctl33$linkEditSearch','')) S6 |
| S5 | TI "accident and emergency" | Search modes - Boolean/Phrase | 887 | [Edit](javascript:__doPostBack('ctl00$ctl00$MainContentArea$MainContentArea$editControl$printHistory$HistoryRepeater$ctl34$linkEditSearch','')) S5 |
| S4 | TI emergency room | Search modes - Boolean/Phrase | 892 | [Edit](javascript:__doPostBack('ctl00$ctl00$MainContentArea$MainContentArea$editControl$printHistory$HistoryRepeater$ctl35$linkEditSearch','')) S4 |
| S3 | TI emergency medicine | Search modes - Boolean/Phrase | 3,948 | [Edit](javascript:__doPostBack('ctl00$ctl00$MainContentArea$MainContentArea$editControl$printHistory$HistoryRepeater$ctl36$linkEditSearch','')) S3 |
| S2 | (MM "Trauma Centers") | Search modes - Boolean/Phrase | 1,604 | [Edit](javascript:__doPostBack('ctl00$ctl00$MainContentArea$MainContentArea$editControl$printHistory$HistoryRepeater$ctl37$linkEditSearch','')) S2 |
| S1 | (MM "Emergency Medicine") | Search modes - Boolean/Phrase | 3,255 | [Edit](javascript:__doPostBack('ctl00$ctl00$MainContentArea$MainContentArea$editControl$printHistory$HistoryRepeater$ctl38$linkEditSearch','')) |
