# Supplementary material for: Managing Acute Behavioural Disturbances in the Emergency Department Using the Environment, Policies and Practices: A Systematic Review
Source: West J Emerg Med. 2017 May 15;18(4):647–61. doi: 10.5811/westjem.2017.4.33411 (PMC5468071; doi:10.5811/westjem.2017.4.33411)
Supplement: Supplementary file 4 [file wjem-18-647-s004.docx]

APPENDIX D: Search undertaken using EMBASE

| 1 | emergency medicine.mp. or Emergency Medicine/ | 41081 | Advanced |
| --- | --- | --- | --- |
| 2 | (emergency department* or ED* or "A&E" or emergency room*).m_titl. | 374194 | Advanced |
| 3 | *emergency health service/ | 40736 | Advanced |
| 4 | 1 or 2 or 3 | 438088 | Advanced |
| 5 | (aggression or difficult or demand* or disrupt* or assault* or threat* or antagonistic or hostile or combat* or confront* or argument or behavio* or challeng*).ti. | 480208 | Advanced |
| 6 | *exposure to violence/ or *workplace violence/ | 313 | Advanced |
| 7 | violence/ or violence.mp. | 63432 | Advanced |
| 8 | workplace violence.mp. or *workplace violence/ | 1117 | Advanced |
| 9 | "violen*".m_titl. | 23690 | Advanced |
| 10 | Agonistic Behavior.mp. | 621 | Advanced |
| 11 | Patient Self-Determination Act/ | 121 | Advanced |
| 12 | 5 or 6 or 7 or 8 or 9 or 10 or 11 | 536960 | Advanced |
| 13 | patient isolation.mp. or Patient Isolation/ | 225342 | Advanced |
| 14 | *Immobilization/ | 7975 | Advanced |
| 15 | patients' rooms.mp. [mp=title, abstract, heading word, drug trade name, original title, device manufacturer, drug manufacturer, device trade name, keyword] | 180 | Advanced |
| 16 | health facility environment.mp. | 34 | Advanced |
| 17 | confined spaces/ | 539 | Advanced |
| 18 | *Environment Design/ | 3208 | Advanced |
| 19 | (isolat* or confine* or hold or restrain* or room* or sensory*).tw. | 1890410 | Advanced |
| 20 | *risk reduction behavior/ | 3488 | Advanced |
| 21 | *Risk Assessment/ | 31207 | Advanced |
| 22 | organizational policy.mp. or Organizational Policy/ | 74422 | Advanced |
| 23 | (policy or management).m_titl. | 425028 | Advanced |
| 24 | 13 or 14 or 15 or 16 or 17 or 18 or 19 or 20 or 21 or 22 or 23 | 2603191 | Advanced |
| 25 | 4 and 12 and 24 | 1359 | Advanced |
| 26 | limit 25 to (english language and yr="1985 -Current") | 1177 |  |
